# Effects of meditation on physiological and metabolic parameters in patients with type 2 diabetes mellitus “MindDM”: study protocol for a randomized controlled trial

**DOI:** 10.1186/s13063-022-06771-2

**Published:** 2022-09-30

**Authors:** K. P. C. Dalpatadu, P. Galappatthy, P. Katulanda, S. Jayasinghe

**Affiliations:** Faculty of Medicine, University of Colombo, No:25, Kinsey Road, Colombo, 8 Sri Lanka

**Keywords:** Mindfulness meditation, Diabetes mellitus, Mechanisms of metabolic control

## Abstract

**Background:**

Sri Lanka is faced with the challenge of managing a large population with diabetes mellitus by 2030. Psychological stress plays a major role in disease outcome by exerting physiological, psychological and social effects on individuals with chronic disorders.

Meditation-based interventions have positive effects on the management of stress and diabetes, which are mediated via modulation of neuro-humoral mechanisms and autonomic functions, among others. Mechanisms of bio-physiological effects of meditation are considered to be through reduction of stress hormones, improvement of insulin resistance and improvement of autonomic dysfunction.

**Methods:**

This study will be conducted as an open-label, randomized controlled clinical trial in the Faculty of Medicine, University of Colombo. The aim is to investigate the effects of meditation on glycaemic control and possible mechanisms of how meditation affects glycaemic control in patients with type 2 diabetes. The study was approved by the Ethics Review Committee of the Faculty of Medicine, University of Colombo (ERC/2019/094). Patients who are attending the professorial unit medical clinic with type 2 diabetes (172 in total) will be recruited based on inclusion-exclusion criteria. Patients who have never meditated or rarely meditated (less than once every three months) will be randomized using block randomization to meditation and waitlisted arms (1:1 allocation ratio). The meditation arm will undergo a mindfulness meditation program (selected after studying several meditation methods) conducted by a qualified instructor weekly for a period of 12 weeks in addition to usual care, while the waitlisted arm will only receive usual care. Daily meditation practices will be recorded in a diary. The primary outcome measure is HbA1c. Secondary outcome measures are fasting blood sugar, fructosamine, insulin resistance (calculated using fasting serum insulin), 24-h urinary cortisol, body mass index, cardiac autonomic reflex testing (Ewing’s battery of tests) and orocecal transit time using hydrogen breath analysis. All these will be done prior to commencement of the intervention and after 3 months in both arms. Data will be analysed using SPSS V-23.

**Discussion:**

This study aims to identify the effect of mindfulness meditation on glycaemic control and the possible mechanisms (neuro humoral and autonomic functions) by which beneficial effects are mediated.

**Trial registration:**

Registered under Sri Lanka Clinical Trial Registry: SLCTR/2021/015

The Universal Trial Number (UTN) U1111-1266-8640

**Supplementary Information:**

The online version contains supplementary material available at 10.1186/s13063-022-06771-2.

## Administrative information

Note: the numbers in curly brackets in this protocol refer to SPIRIT checklist item numbers. The order of the items has been modified to group similar items (see http://www.equator-network.org/reporting-guidelines/spirit-2013-statement-defining-standard-protocol-items-for-clinical-trials/)

**Table:** 

**Title {1}**	Effects of meditation on physiological and metabolic parameters in patients with type 2 diabetes mellitus.”MindDM trial
**Trial registration {2a and 2b}.**	Registered under Sri Lanka Clinical Trial Registry : SLCTR/2021/015 The Universal Trial Number (UTN) U1111-1266-8640
**Protocol version {3}**	Protocol version 3.0(26.10.2020)
**Funding {4}**	World bank-Accelerating Higher Education Expansion and Development (AHEAD) grant-81
**Author details {5a}**	I. Dr. K.P.C Dalpatadu-Corresponding author chamila@physiol.cmb.ac.lk Senior Lecturer in Physiology, Faculty of Medicine, University of Colombo II. Professor P. Galappatthy p.galappatthy@pharm.cmb.ac.lk Professor of Pharmacology, Faculty of Medicine, University of Colombo III. Professor P. Katulanda Prasad.katulanda@clinmed.cmb.ac.lk Professor in Medicine, Faculty of Medicine, University of Colombo IV. Professor S. Jayasinghe saroj@clinmed.cmb.ac.lk Emeritus Professor in Medicine, Faculty of Medicine, University of Colombo Institutional address-No:25, Kinsey Road, Colombo 8, Sri Lanka Number 25,
**Name and contact information for the trial sponsor {5b}**	World Bank AHEAD grant scheme awarded to University of Colombo, Sri Lanka. AHEAD operations, 79/1, 5^th^ Lane, Colombo 03, Sri Lanka. Tel +94112432980
**Role of sponsor {5c}**	Study sponsor does not have authority over study design; collection, management, analysis, and interpretation of data; writing of the report; and the decision to submit the report for publication

## Introduction

### Background and rationale{6a}

The prevalence of diabetes mellitus is rising worldwide, especially in the Southeast Asian region [1] and in Sri Lanka it has been projected to rise up to 13.9% by 2030 [2].

In both health and disease, psychological stress plays a major role in determining the well-being of an individual. It affects the individual’s behaviour pattern, biophysical parameters and how the individual copes with disease [3]. It has been postulated that physiological processes are affected by stress through the neuro-endocrine response system. Stress stimulates both the hypothalamic-pituitary-adrenocortical axis and the sympathetic-adrenal-medullary system, which in turn affects a wide range of physiological processes [3]. Chronic stress leads to elevated cortisol levels and results in poor glycaemic control [4] It also indirectly affects glycaemic control through changes in behaviour patterns such as diet and exercise [5]. It has been recognized that stress has a negative effect on quality of life and psychosocial wellbeing [3] in patients with diabetes.

Meditation can be defined as the intentional self-regulation of attention from moment to moment [6]. The roots of most meditation practices have evolved from Buddhism. There are two major areas in meditation: concentration meditation and insightful meditation. Concentration meditation is known as “*Samatha*” in Theravada Buddhism and involves focussing attention to one thing [6]. Insightful or awareness meditation, which is known as “vipassana” in Theravada Buddhism, does not restrict attention to one thing but emphasizes detached self-observation from one moment to another [6]. However, these two areas are not mutually exclusive and can be described as two aspects of the same meditative state [7]. It has also been viewed as “*Samatha*” meditation practices leading to stability of the mind, whereas “*Vipassana*” meditation practices leading to clarity of the mind [7]. Furthermore, a state of stability of the mind is needed to achieve clarity of mind. It is important to understand that Buddhist meditation is not merely a method of relaxation but is aimed at achieving a balance between stability and clarity of mind through practice [7]. The meditative state thus reached is said to be present not only during meditation but also after meditation. The modern term of mindfulness or “*Sati*” in Buddhist literature, is described as a state of mind achieved through focussing the attention on one thing and being in the present moment. This paves the pathway to achieve a higher state of mind through meditation. The aim is to have focused attention as well as to identify when the attention is deviated and regained [7]. One example is “*Anapanasathi*” meditation which has been described as keeping the focused attention on breathing and being at the present moment (breath awareness).

In clinical psychology, meditation is defined as “A family of self-regulation practices that focus on training attention and awareness in order to bring mental processes under greater voluntary control and thereby foster general mental well-being and development and/or specific capacities such as calm, clarity, and concentration” [8]. Thus, the core components of meditation practices include focused attention, self-regulation and self-awareness [8].

There is evidence to suggest that meditation-based interventions have positive effects on the management of stress and diabetes [3, 4, 9, 10]. The beneficial effect of meditation on the management of diabetes includes; (a) modulation of neuro-humoral mechanisms that are involved in the management of stress, (b) improvement of overall coping ability with a chronic illness and (c) favourable impact on behaviours such as compliance with diet control, exercise regimen and medications. The development of present moment awareness promotes non-judgmental acceptance of the present status and encourages individuals not to indulge in ruminative thoughts on previous or anticipated events [3]. This aspect of meditation helps the individual to come to terms with chronic illnesses such as diabetes. Therefore, mindfulness meditation practices can be viewed as techniques that sharpen the awareness and bring peace and tranquility. These can also be viewed as mindfulness-based intervention or mind-body therapy.

It has been revealed that walking meditation, a form of concentration meditation, produces multiple favourable effects, such as a reduction in cortisol levels, better glycaemic control and reduced arterial stiffness in patients with type 2 diabetes [4]. Rosenzweig et al. [9] analysed the effects of mindfulness-based stress reduction therapy (MBST) on measures of HbA1c, blood pressure, body weight, and psychological symptoms in patients with diabetes. This pilot study found a reduction in HbA1c of 0.5%, reduction in mean arterial pressure of 6 mmHg, and reduction in depression and anxiety scores.

Researchers have identified the regions of the brain involved during various aspects of meditation [11]. For example, the anterior cingulate cortex and striatum in attention control, multiple prefrontal regions, limbic regions and striatum in emotion regulation and insula, medial prefrontal cortex posterior cingulate cortex and precuneus in self-awareness [11]. The metabolic effects of meditation are through neuro-humoral modulation and stress reduction. Even short-term meditation has been shown to increase the activity of the prefrontal cortex, which is involved in stress regulation. Meditation suppresses the activity of the amygdala and anterior cingulate cortex, which are involved in flight or fight reaction [11] thus reducing stress hormone levels.

In a study that assessed neural activity associated with “*Anapanasathi*” meditation and love and kindness meditation (*Metta*), it was noted that after 30 min of meditation, significant changes in functional MRI signals were observed in the right middle frontal gyrus, left inferior parietal lobe, bilateral middle temporal gyrus, bilateral superior temporal gyrus, right inferior temporal gyrus, and left middle occipital gyrus both in novices and experienced meditators [12]. These findings are similar to the other studies performed in long-term meditators [11]. Thus, it can be hypothesized that the relaxation response thus brought about through meditation leads to beneficial effects such as improvement of glycaemic control through mechanisms such as reduction of stress hormone levels.

Furthermore, it has been found that mind body therapies such as yoga, tai chi, qigong, and meditation may supplement the conventional care and management of metabolic syndrome [13].

Meditation exerts changes in autonomic functions as well. In an Indian study, it was shown that parasympathetic tone was significantly higher in the meditators than in the control group [14]. As autonomic neuronal dysfunction is related to diabetes control and many complications of diabetes (gastrointestinal dysfunction and cardiac autonomic dysfunction), it will be useful to investigate the changes in autonomic functions, in patients with diabetes when meditation is practised.

Intestinal dysmotility due to diabetic autonomic neuropathy [15] and diabetic enteric neuropathy has been implicated as the main reason for gastrointestinal complications such as small intestinal bacterial overgrowth [16], gastro-paresis, slow-transit constipation and diabetic diarrhoea. These complications lead to poor glycaemic control owing to erratic absorption of glucose [15]. Stress has been shown to increase gastric emptying time, increase distal colonic motility and accelerate small intestinal transit [17]. It is possible to hypothesize that psychological stress-induced changes in intestinal motility have a negative impact on glycaemic control due to erratic glucose absorption even in the absence of diabetes-related autonomic neuropathy and enteric neuropathy.

The effects of cardiac autonomic neuropathy, such as reduced heart rate variability during deep breathing, decreased baroreflex sensitivity, and orthostatic hypotension [18], can be assessed noninvasively, by 5 simple tests (Ewing et al.) [19]; cardiac autonomic reflex testing (CART) [19]. However, the heart rate response to deep breathing is the test that is most frequently utilized because of its high reproducibility and specificity [20].

There is evidence from a small study involving healthy adults that meditation causes an increase in heart rate variability and causes a decrease in the low-frequency to high-frequency ratio (more predominant vagal tone), which indicates improved autonomic function [21]. Furthermore, it has been shown that, in diabetic cardiac autonomic neuropathy, stress decreases high-frequency heart rate variations [22]. Thus, it can be hypothesized that the stress-relieving effect of meditation will have an effect on reduction of low-frequency to high-frequency heart rate variations, indicating better autonomic function. Improvement of cardiac autonomic testing can be taken as a marker of improvement of overall autonomic functions, which might have beneficial effects on the sympathetic-adrenal medullary system, thus improving the glycaemic control.

Thus, it can be hypothesized that the mechanisms underlying the bio-physiological effects of meditation would be through reduction of stress hormones (cortisol), improvement of insulin resistance and improvement of autonomic dysfunction [3].

Therefore, we report the original protocol of the study aiming to identify the impact of meditation on glycaemic control and the possible mechanisms, such as modulation of the autonomic nervous system, reduction of cortisol levels, improvement in gut transit time and improvement in insulin resistance.

To date, there had not been any clinical trial conducted in Sri Lanka studying the effects of meditation in patients with diabetes. The acceptability of meditation is likely to be high in Sri Lanka, with a population of over 70% Buddhists (Department of Senses and Statistics 2012). With the growing population of diabetes in Sri Lanka, exploration of the effects of mind-body therapies such as meditation is timely, as it is cost-effective, non-invasive and has minimal negative effects. If proven effective, in the future, meditation can be successfully incorporated into the comprehensive management of patients with diabetes to improve physical-psychological and social health in the Sri Lankan setup with minimal resources.

### Objectives {7}

We hypothesize that improvement occurs in selected metabolic and physiological parameters in patients with type 2 diabetes following meditation, independent of standard care. We also hypothesize that these effects occur due to the reduction of stress hormones (cortisol), improvement of insulin resistance and improvement of autonomic dysfunction.

The general objective is to determine the effects of meditation on selected metabolic and physiological parameters in patients with type 2 diabetes by conducting an open-label randomized controlled clinical trial and investigating the possible mechanisms by which meditation affects these parameters.

Specific objectives are to determine the effects of meditation on glycaemic control, lipid profile and blood pressure, insulin resistance, 24 urinary cortisol, orocecal transit time using lactulose hydrogen breath test and cardiac autonomic functions. This will be done using standard cardiac autonomic reflex testing (CART), and further analysis of ECG recording in order to analyse heart rate variations(HRV) using Power Lab-8 Pro software.

## Methods: Participants, interventions and outcomes

### Trial design {8}

Open-label randomized control parallel group explanatory clinical trial. It will be conducted on patients with type 2 DM who are attending the professorial medical unit clinic at the National Hospital of Sri Lanka (NHSL). Patients will be randomized to meditation and waitlisted arms after an interview and obtaining informed consent. Meditation intervention and all investigations will be carried out in the Department of Physiology, Faculty of Medicine, University of Colombo.

### Study setting{9}

The study will be conducted in a single centre; the National Hospital of Sri Lanka (NHSL), which is a tertiary care hospital. Patients with type 2 diabetes who are attending the professorial unit (academic unit) medical clinic at NHSL are invited to participate in this study. All physiological testing will be carried out in the Department of Physiology, Faculty of Medicine, University of Colombo.

### Eligibility criteria{10}

Adult patients diagnosed with type 2 diabetes within the past 3-5 years who have never meditated before or rarely meditated (less than once in three months) will be recruited to meditation and waitlisted arms, according to the following inclusion and exclusion criteria.

i. Age between 18 and 75 years
ii. Willingness to participate in a meditation program
iii. Commitment to adhere to the program for 3 months.
iv. Patients taking ≤ 2 anti-diabetic medications
v. HbA1c between 7 and 8%
vi. Education level secondary level and above (grade 8 and above)
vii. Montreal Cognitive Assessment Score more than 26/30

Exclusion criteria

i. Long-term meditators/experienced meditators.
ii. Hemoglobinopathy that can interfere with HbA_1_c monitoring
iii. Renal impairment
iv. Any malignancy
v. Any other chronic disorders (ex. thyroid disease)
vi. Patients on steroids, insulin, beta-blockers, calcium channel blockers cisapride, domperidone, other anti-arrhythmic medications, antidepressants, antihistamines and sympathomimetic cough preparations [23]
vii. Alcohol abuse or use of neurotoxic medication
viii. History of posttraumatic stress disorder, epilepsy, depression or psychosis.
ix. Autonomic dysfunction or peripheral neuropathy was diagnosed.
x. Presence of acute illness and severe systemic disease
xi. Angina pectoris
xii. Congestive heart failure NYHA Class III–IV
xiii. Diagnosed cardiac arrhythmia
xiv. Myocardial infarction or invasive cardiovascular procedure within the past six months.
xv. Stroke in the last 6 months
xvi. Patients with chronic diarrhoea
xvii. Patients who are unable to attend weekly meditation sessions
xviii. Patients not consenting to meditation
xix. Montreal Cognitive Assessment Score of less than 26/30

### Who will take informed consent? {26a}

Informed written consent will be obtained from the participants by the investigators using a consent form after explaining and providing the information sheet.

### Additional consent provisions for the collection and use of participant data and biological specimens {26b}

On the consent form, participants will be asked if they agree to give biological specimens (blood) for the investigations that are explicitly mentioned. Should they choose to withdraw from the trial, data will be used for analysis only if they give permission. Participants will also be asked for permission for the research team to share relevant data, excluding any personal data when publishing the results.

## Interventions

### Explanation for the choice of comparators {6b}

The meditation technique for the study was selected after observation and discussions held on several different meditation techniques used by long-term meditators. Planned intervention is a simple technique that can be grasped by the absolute novice. It is acceptable to patients of any religion and leads to tranquility of mind through techniques such as awareness of one’s own respiration [24]. Meditation intervention includes mindful walking meditation and mindful breathing. This meditation technique has been used by the consultant psychiatrist (meditation instructor of our study) among his patients for a long time.

### Intervention description {11a}

The meditation arm will undergo a mindfulness meditation program aiming to achieve calmness and tranquility of mind under the guidance of an experienced instructor for a period of 12 weeks (protocol of intervention-Additional file 1) in addition to usual care. This is a complex intervention and follows the 2006 updated Medical Research Council guidelines. The waitlisted arm will only receive the usual care provided by the clinic. The waitlisted arm will undergo the intervention in the second 3 months, while the meditation arm will be followed up without active meditation intervention.

The adherence to the meditation routine while the patient is not actively monitored will be recorded.

#### Ethical aspects of the intervention used

Although mindfulness meditation is related to Buddhist philosophy, the techniques used in this clinical trial do not involve any Buddhist teaching. However, when non-Buddhist patients are recruited even after their consent, there may be consequences such as pressure from their religious leaders, family members and peers. This may result in higher rates of dropouts, which is a drawback in these types of studies where the invention does have a religious background. If such instances occur, investigators are dedicated to support their decision to leave the study if desired and to maintain confidentiality at all times.

### Criteria for discontinuing or modifying allocated interventions {11b}

The study will be terminated if there are more psychological/behavioural adverse events reported in the meditation group than in the control group or if there is more than 60% drop-out rate from the meditation program. A strong request from participants will be another reason for termination.

### Strategies to improve adherence to interventions {11c}

Participants will be given a diary to record the meditation practices done at home, which will be checked at each session, and instructions will be given. This will include a record of time spent meditating, problems that occurred, doubts, reasons for distraction, etc. Participants are expected to record their meditation practice daily on most days of the week between weekly sessions. The diary will be used as the tool to check compliance with the intervention.

### Relevant concomitant care permitted or prohibited during the trial {11d}

Concomitant care apart from meditation intervention in both arms will be routine care during monthly clinic visits, which includes medication review and advice, dietary advice, referral to a dietician, and screening for neuropathy and retinopathy. All participants are prohibited from practising in any other form of mind-body therapies, such as yoga.

### Provisions for posttrial care {30}

Patients will have the ability to contact the investigators for any clarifications throughout the study and after the completion of the intervention for a period of one year. Patients will be followed up in their monthly clinic with routine care after the trial. In addition, during the period of the meditation program, they can contact the instructor. They are free to contact either the investigators or the ERC to lodge complaints regarding the study. Any change in the protocol will be communicated to the participants after ethical clearance and consent will be retaken for the study.

### Outcomes {12}

The primary outcome is to study the effect of meditation on glycaemic control in patients with type 2 diabetes as measured by HbA1c.

#### Secondary outcomes

- The effect on short-term and intermediate blood sugar control as measured by FBS and fructosamine
- The effect of meditation on lipid profile.
- The effect of meditation on blood pressure.
- Analysis of the following possible mechanisms leading to changes in glycaemic and metabolic control in each arm (insulin resistance, autonomic functions, gut transit and 24-h urinary cortisol)

### Participant timeline {13}

Each investigation will be carried out at baseline and postintervention after 12 weeks in all patients in both the intervention and waitlisted arms. Details of the study schedule are given in Table 1.

**Table 1:** Study schedule

Study period						
Assessments	Time point					
	Enrolment Allocation	Baseline assessments	Intervention weekly for 12 weeks	Post intervention visit at 3months	Intervention weekly for 12 weeks	Visit at 6 months
Eligibility screen	*					
Montreal cognitive assessment (MOCA)	*					
Informed consent	*					
Random allocation	*					
Meditation intervention			*(meditation arm only)		*(waitlisted arm only)	
**Assessments**						
Medical history/Interviewer administered data collection	*					
Fasting blood sugar		*	*(monthly at clinic)	*	*(monthly at clinic)	*
HbA1c		*		*		*
Fructosamine		*		*		*
Fasting serum insulin		*		*		*
Full blood count		*				
Liver /renal functions^a^		*				
24-h urinary free cortisol		*		*		*
Lipid profile		*		*		*
Body mass index		*		*		
Blood pressure		*	*(monthly at clinic)	*	*(monthly at clinic)	*
Autonomic function tests		*		*		*
Oro-caecal transit time		*		*		*

^a^Renal functions (serum creatinine, serum electrolytes), liver functions (AST/ALT)

### Sample size {14}

Patients with type 2 DM will be randomized to meditation and waitlisted arms. The number of patients required was 86 in each arm, which was calculated on the basis of determining a 0.5% reduction in HbA1c in the meditation arm in comparison to the waitlisted arm. The basis of 0.5% reduction is derived from looking at several randomized controlled trials (RCT) which have shown a significant reduction of HbA1c in the meditation arm compared to control arm. The reduction of HbA1c were; 0.5% in Gainey et al. 2016 [4], 0.7% in Gregg et al. 2007 [25], 0.74% in Pearson et al. 2018 [26], 0.83% in Miller et al 2012 [27], 1.0% in Armani Kian et al. 2018 [28], 1.1% in Chen et al. 2020 [29]. According to a meta-analysis, most of the oral hypoglycaemic drugs lowered HbA1c by 0.5% [30] as well. Therefore, the effect size was taken as a 0.5% difference in the reduction of HbA1c indicating that the lowest significant reduction of HbA1c is 0.5%. A power of 70% and a 95% confidence interval with a drop-out rate of 20% were considered.

The following formula was used for sample size calculation:

$$N=\left[\left(1/{q}_1+1/{q}_0\right)\ast {\left({Z}_{\alpha }+{Z}_{\beta}\right)}^2\right]/\left[{\left(E/S\right)}^2\right]$$

where

*N* = Sample size

*q*1 = Proportion of subjects in the meditation group

*q*2 = Proportion of subjects that are in the waitlisted group

*Z*_*α*_ = Critical value of the normal distribution at *α* (*α* is 0.05 and the critical value is 1.96)

*Z*_*β*_ = Critical value of the normal distribution at *β* (*β* is 0.3)

*E* = Effect size (0.5% difference in HbA1c between the two groups)

*S* = Standard deviation of HbA1c in the population (0.8%).

### Recruitment{15}

Patients with type 2 diabetes who are attending the professorial unit medical clinic at the National Hospital of Sri Lanka (NHSL) will be invited to the study. An eligibility screen and a Montreal cognitive assessment (MOCA) will be performed in participants who volunteer. They will be randomized to meditation and waitlisted arms after obtaining informed written consent.

## Assignment of interventions: allocation

### Sequence generation {16a}

Patients will be randomized to meditation and waitlisted arms with a one-to-one allocation ratio using block randomization of 10 according to an online random number generation program.

### Concealment mechanism {16b}

Sealed envelopes will be prepared in advance, indicating the allocation of each patient according to the recruitment number. At the time of recruitment, the envelopes indicating the allocation will be provided to the researchers who will enrol patients. Thereafter, those who satisfy inclusion and exclusion criteria and give informed consent, will be assigned a unique patient recruitment number in chronological order. The sealed envelope with the allocation for the given recruitment number is opened, and accordingly patients will then either start the meditation programme or be allocated to the waitlisted arm.

### Implementation {16c}

A member of the study team not involved in patient recruitment will generate the allocation sequence for assigning participants according to generated random numbers.

## Assignment of interventions: blinding

### Who will be blinded {17a}

Data analysts and laboratory staff analysing samples and performing tests will be blinded to the allocation. The trial participant and the investigators will not be blinded due to the nature of the intervention.

### Procedure for unblinding if needed {17b}

Unbinding will only be done if there is a need to further analyse blood samples in case of erroneous results.

## Data collection and management

### Plan of assessment and collection of outcomes{18a}

The study will be conducted for a period of 6 months. Assessments will be conducted in the following manner at recruitment: screening (baseline), 3 months, and 6 months in both the intervention and waitlisted groups.

#### Measurement tools

##### Anthropometric measurements

BMI: Height is measured to the nearest 0.1 cm (maximum distance from heels to uppermost position on head while standing barefoot in full inspiration using a stadiometer).

Body weight is measured to the nearest 0.1 kg using a digital scale (Secca) while wearing indoor light clothing. BMI is calculated as weight in kg/height in m^2^.

##### Clinical examination

Blood pressure: blood pressure in the seated position will be measured after 10 min of rest using a Riester digital blood pressure monitor (Rudolf Riester, Germany). Blood pressure will be checked twice.

##### Laboratory investigations

FBS, HbA1c, lipid profile, fructosamine, fasting serum insulin and 24-h urinary free cortisol will be measured in an accredited laboratory. Fasting plasma glucose will be measured in a fully automated biochemistry analyser (hexokinase method), and the lipid profile will be measured by an enzymatic method. HbA1c will be analysed using a high-performance liquid chromatography (HPLC) method. Insulin resistance will be calculated with the homeostasis model assessment (HOMA) equation: fasting glucose (mg/dL)×insulin level (uU/mL)/405 [4].

##### Orocecal transit time

The gold standard method to assess gastric emptying is radioisotope scintigraphy [31]. However, due to the noninvasive and simple technique involved, we opted for the orocecal transit time(OCTT) assessment using a hydrogen breath analyzer. Furthermore, by using OCTT, in addition to gastric emptying we can assess small bowel motility, which has an impact on glucose absorption and thus glycaemic control. Although there are more advanced newer techniques, such as MRI scans and capsular endoscopy [32], to assess OCTT, due to the high cost, these tests cannot be used in our study.

#### Lactulose hydrogen breath test

The time interval between ingestion of test meal containing 10 ml of lactulose after overnight fast and rise in breath hydrogen 10 ppm above basal using hydrogen breath analyser (Gastrolyzer, Bedfont Scientific Ltd, UK) is a measure of orocecal transit time (OCTT) [33, 34].

A solution including 10 g of lactulose in 100 mL of water will be administered. The breath test is performed at 10-min intervals, and a rise in hydrogen concentrations ≥10 ppm (particles per million) compared to baseline followed by at least two other subsequent rises are taken as OCTT. The OCTT in healthy subjects is between 40 and 170 min for a liquid diet [35].

##### Cardiac autonomic reflex assessment: CART

Autonomic functions will be assessed using Human Physiology AFT equipment (PL2604 by AD Instruments Pvt. Ltd, Australia)

- Heart rate response to deep breathing, which assesses beat-to-beat *R*-*R* variation during six breaths per minute paced slow deep breathing [expiration-to-inspiration ratio (*E*:*I*)=*R*-*R*_max_/*R*-*R*_min_].
- Heart rate response to standing is expressed as the ratio between the longest R-R interval (between the 20th and 40th beats) to the shortest R-R interval (between the 5th and 25th) after standing from the lying position [23].
- Valsalva manoeuvre and calculate the Valsalva ratio (maximum heart rate in phase II: minimal heart rate in phase IV)
- The blood pressure response after 3 min of standing
- The blood pressure response to sustained hand grip

We will be recording a 15-min of resting ECG before the standard CART protocol. From which a 5-min recording [36] after the patient stabilizes, will be used for subsequent heart rate variation (HRV) analysis.

Spectral analysis of HRV, both time domain analysis and frequency domain analysis of the 5-min resting ECG tracing, will be performed using a Power Lab 8 data acquisition system [37]. The following parameters will be assessed according to the Task Force of the European Society of Cardiology and the North American Society of Pacing and Electrophysiology guidelines(1996) [38].

In time domain analysis, SDNN (standard deviation of normal-to-normal RR intervals), SDANN (standard deviation of the average normal-to-normal intervals for each 5-min segment of the ECG recording), SDNNI (mean of the standard deviation of all the normal-to-normal intervals for each 5-min segment of the recording), and RMSSD (root mean square of successive RR interval differences) will be analysed. In frequency-domain analysis, VLF (absolute power of the very-low-frequency band: 0.0033–0.04 Hz), LF (absolute power of the low-frequency band: 0.04–0.15 Hz), HF (absolute power of the high-frequency band: 0.15–0.4 Hz) and LF:HF (ratio of LF-to-HF power) [38] will be analysed. Pre- and post-meditation HRV analysis as well as any difference between the intervention and control arms will be compared. Furthermore, it has been shown that the cardiac vagal tone assessed using 5-min resting electrocardiogram recording has a good correlation with HRV analysis performed with 24-h recording in detecting subclinical cardiac autonomic neuropathy in patients with type 1 diabetes [39].

### Plans to promote participant retention and complete follow-up {18b}

Patients are given access to contact the meditation trainer and the investigators throughout the trial period to clarify doubts. Primary outcome data will be collected in patients who discontinue or deviate from the intervention protocol if consent is granted. Participants will be contacted by the investigators periodically and encouraged daily practice of meditation and documentation in the diary.

### Data management {19}

Data will be entered by a dedicated person who is blinded to the patient allocation. Double data entry will be performed and will be stored under password protection in a dedicated computer.

### Confidentiality{27}

All interviews will take place in a confidential room. Personal information is only gathered to ensure the correct identification of patients when issuing the investigation reports. It will not be shared with a third party without their consent, and all data gathered will be securely kept under the direct supervision of the investigators. All data gathered will be stored securely for 5 years and then destroyed. Only the principal investigator and supervisors will have the authority to access the data.

### Plans for collection, laboratory evaluation and storage of biological specimens for genetic or molecular analysis in this trial/future use {33}

All the blood samples taken will only be used for the intended test, and no genetic studies will be performed. Blood samples will not be sent abroad or given to a third party. All samples will be stored for a period of one month for the completion of the tests and securely destroyed. All the reports of the blood tests will be given to the patient with a copy kept with the investigators.

## Statistical methods

### Statistical methods for primary and secondary outcomes {20a}

Statistical analysis will be performed using SPSS v-23. Intention to treat analysis will be carried out. Data will be analysed using descriptive statistics (frequency distribution, mean) to demonstrate the distribution of data for each parameter. Comparative statistics will be used to compare the findings between the sample groups, within the sample and control. Correlation analysis will be conducted to assess the correlation between related parameters. Each of the outcomes will be analysed using multiple regression analysis to ascertain the differences between trial arms. A linear regression model for continuous variables and logistic regression for binary variables will be used.

### Data monitoring committee{21a}

An independent data safety monitoring committee was appointed comprising senior researchers who have no competing interests. Any adverse events occurring will be reported to the committee by investigators and will be independently assessed.

### Interim analyses {21b}

If serious adverse events related to the intervention are reported in the meditation arm, the data safety monitoring committee and the investigators will discuss and decide to terminate the trial. Thereafter, an interim analysis will be conducted from the available data. The ethics review committee and the Sri Lanka Clinical trial registry will have access to interim results.

### Methods for additional analyses (e.g., subgroup analyses) {20b}

Subgroup analysis of the primary and secondary outcomes will be performed according to the age, gender, duration of diabetes and baseline HbA1c.

### Methods in analysis to handle protocol nonadherence and any statistical methods to handle missing data {20c}

We are planning to conduct an intention-to-treat analysis. Therefore, primary outcome data of the participants randomized to the intervention but who did not adhere to the intervention will be imputed using multiple imputation methods [40] available in SPSS.

### Plans to give access to the full protocol, participant-level data and statistical code {31c}

The datasets analysed during the current study and statistical codes are available from the corresponding author on reasonable request, as is the full protocol.

## Oversight and monitoring

### Composition of the coordinating centre and trial steering committee {5d}

The coordinating centre will be the Department of Physiology, Faculty of Medicine, University of Colombo, led by the Head of the department. The trial steering committee will comprise of investigators and medically qualified research assistants. The role and responsibility of the coordinating centre and trial steering committee will be, to coordinate patient recruitment, provide support with coordinating appointments for investigations, provide day-to-day liaison service and solve technical issues. The steering committee will closely monitor technical officers regarding maintenance of equipment and supply of consumables. The research promotion and facilitation centre (RPFC) of the Faculty of Medicine, University of Colombo, will provide oversight. The trial steering committee will meet once a month over the course of the trial to discuss the progress.

The progress report will be submitted periodically to the ethics review committee and research and higher degrees committees.

### Adverse event reporting and harms {22}

Patients will also be monitored throughout the study for any adverse events related or unrelated to meditation (e.g., adverse effects of anti-diabetic drugs). Their health and well-being will be assessed through clinical assessment and monitoring of investigations. Serious adverse events will be monitored by the data safety monitoring board and reported to the ethics committee and the treating physician. The necessary actions will be taken, including further investigations and management steps. Patients who suffer any serious adverse events will be monitored and followed up for an extended period of 2 years, regardless of whether they continue to be part of the study.

### Frequency and plans for auditing trial conduct {23}

The Project Management Group, comprising both the members from the coordinating centre and trial steering committee, will meet monthly to review the trial conduct. The trial Steering committee and the independent data safety monitoring board will meet monthly and if there are any issues reported to the Ethics Review Committee of the Faculty of Medicine, University of Colombo, throughout the trial period. Any protocol amendments will be duly notified to the Ethics Review Committee, and approval will be taken prior to implementation.

### Plans for communicating important protocol amendments to relevant parties(e.g., trial participants, ethical committees) {25}

Any amendments to the protocol will be communicated in writing to all participants, and consent will be taken from them prior to conduct. Approval for any protocol amendments will be taken from the Ethics review committee of the Faculty of Medicine Colombo and Sri Lanka Clinical trial registry.

### Dissemination plans{31a}

The individual results of the investigations will be shared with the relevant participant. The overall results of the trial will be published in peer review local and international journals and will be presented in scientific conferences. All investigators will be authors, and professional writers will not be involved.

## Discussion

We believe that this will be the first randomized controlled trial in Sri Lanka to assess the effectiveness of mindfulness meditation on the glycaemic control of type 2 diabetes. Although similar studies have been performed in other parts of the world, data from randomized control trials involving South Asians are lacking. As meditation intervention may have different outcomes in different populations, the results from our study will benefit the management of a growing population of patients with diabetes mellitus in our region. Furthermore, some of the possible mechanisms of how meditation brings about glycaemic control, such as its effect on autonomic function and gut transit time, will be studied for the first time in this trial. However, it is difficult to assess the generalizability and fidelity of the intervention across all patients as meditation is a highly individualised intervention which depends on multitude of factors. This is a shortcoming of this type of a clinical trial.

## Trial status

Trial status- Participant recruitment stage. The planned participant recruitment end date is the 30th of June 2023

## Supplementary Information

**Additional file 1.** Protocol for the meditation intervention

**Additional file 2.**

**Additional file 3.**

**Additional file 4.**

## Data Availability

Only deidentified data will be shared after a written request made to the investigators, and only for meta-analysis following 3 months after publication. The public will be able to access the protocol.

## Abbreviations

DM: Diabetes mellitus
IDF: International Diabetes Federation
MBST: Mindfulness-Based Stress Reduction Therapy
MRI: Magnetic resonance imaging
FBS: Fasting blood sugar
S. Cr: Serum creatinine
ALT: Alanine transaminase
AST: Aspartate transaminase
ESR: Erythrocyte sedimentation rate
FBC: Full blood count
CART: Cardiac autonomic reflex testing
BMI: Body mass index
OCTT: Orocecal transit time
NHSL: National Hospital of Sri Lanka
NYHA: New York Heart Association
HRV: Heart rate variations
ECG: Electrocardiogram
MOCA: Montreal Cognitive Assessment
