# Supplementary material for: Effects of meditation on physiological and metabolic parameters in patients with type 2 diabetes mellitus “MindDM”: study protocol for a randomized controlled trial
Source: Trials. 2022 Sep 30;23:821. doi: 10.1186/s13063-022-06771-2 (PMC9523920; doi:10.1186/s13063-022-06771-2)
Supplement: Supplementary file 1 — Additional file 1. Protocol for the meditation intervention [file 13063_2022_6771_MOESM1_ESM.docx]

**Additional file 1**

Protocols of intervention. Intervention will be done under the direct supervision of the qualified instructor (Consultant psychiatrist) and the principal investigator.

Sub topics

1. **Intervention**

**Mindfulness Meditation protocol**

- Mindfulness Meditation program for a period of 3 months.
- This program is based on Buddhist teaching but the actual program does not have any content of the Buddhist preaching nor will they be asked to restrict normal pattern of behaviour.
- Aim is for the participants to practice mindfulness (keeping the attention in the present moment) techniques and achieve a state of calmness and relaxation of the mind.
- Patients will be expected to attend initially once a week 45-60 minutes sessions at the Department of Physiology, Faculty of Medicine, Colombo for 12 weeks where mindfulness meditation will be taught.
- There will be one-hour introductory lecture which will be followed by personal interview with the experienced instructor
- The techniques used in this mindfulness meditation program are not complex techniques. Participants will be asked to first walk for 5 minutes keeping the attention only to the feet/sensation of the feet. If they get distracted they are expected to again focus on the present moment that is walking.
- Then they will be asked to sit on a chair mindfully (focusing on the posture of your body) for 10 minutes.
- In Mindful breathing they will be asked to focus the attention on to the tip of the nose or the chest where they can feel their breath while quietly breathing.
- They will be asked to focus the attention to the feel of the breath calmly and to feel inhalation and exhalation of breath for 15-20 minutes.
- Several other techniques to focus attention will be also will be taught practiced. Examples listening to a bell, mindful eating, etc.
- After each session the instructor will individually talk to the participants and ask them to describe the mindfulness meditation technique they learnt and clarify any questions. During the interview with the instructor they will be assessed with regards to understanding of the technique and if they were mindful while practicing mindfulness meditation.
- Once weekly sessions will be held for batches of 20 participants in the Department of Physiology, Faculty of Medicine Colombo.
- They will be given home assignments which will only take 30 minutes each day to practice. Participants are expected to do them daily from the beginning for the 12 weeks’ period.
- This is to reinforce and continue practicing of mindfulness meditation at home.
- Participants will be given a diary to record the meditation practices done at home which will be checked at each session and instructions will be given. This will include a record of time spent meditating, problems which occurred, doubts, reasons for distraction etc. Participants are expected to record their meditation practice daily on most days of the week in between face to face sessions. The diary will be used as the tool to check the compliance with the intervention.
- Participants are encouraged and given opportunity to ask questions/clarify any doubts during the training sessions which includes the queries documented on their diaries.
- If any participant wants to further practice meditation or need any clarifications, they will be guided by ta Rev. thero
- If they feel they are not able to participate for the sessions or find it difficult to carry out these techniques even after instructors help, they may leave the study without any consequences.
- They will be given contact details of the instructor on the first day if they need to contact and get more details any clarifications.
