# Supplementary material for: Effects of meditation on physiological and metabolic parameters in patients with type 2 diabetes mellitus “MindDM”: study protocol for a randomized controlled trial
Source: Trials. 2022 Sep 30;23:821. doi: 10.1186/s13063-022-06771-2 (PMC9523920; doi:10.1186/s13063-022-06771-2)
Supplement: Supplementary file 2 — Additional file 2. [file 13063_2022_6771_MOESM2_ESM.docx]

**Physiological tests carried out in the Department of Physiology, Faculty of Medicine Colombo**

These tests will be done under direct supervision of Consultant Physician Dr. Chamila Dalpatadu.

1)Date and time of the appointment_________________________

**Measurement of BMI**(Body mass index)

Your height and weight will be measured.

**Cardiac autonomic reflex assessment**

Autonomic functions will be assessed using Human Physiology AFT equipment (PL2604 by AD Instruments Pvt. Ltd, Australia)

You are requested to refrain from strenuous physical exercise in the 24 hours prior to the appointment given for testing and avoid smoking, eating or drinking coffee consumption for at least 2-3 hours prior to testing. The following will be checked with regards to your function of the heart.

1. Resting heart rate recording will be done for 15 minutes during rest in lying position.
2. Heart rate response to deep breathing while lying , which assesses beat to beat variation during six breathing . You will be asked to take slow deep breaths 6/minute.
3. Your blood pressure response is checked from lying to standing and after 3 minutes of standing.
4. Heart rate response to standing from lying position will also be checked.
5. The Valsalva maneuver which assesses the heart rate response during and after blowing in to the syringe against pressure in seated position.

- You will get to practice at least once before the procedure.
- After approximately 1 minute of relaxation you will be given instruction, “Take deep breath and blow into the syringe. Keep the pressure indicated in the pressure meter at 40 mm Hg for 15 seconds”.
- You will be given feedback on how many seconds left or if the pressure is suboptimal to adjust.
- After waiting for 3 minutes, you will be asked to repeat the maneuver twice, in total 3 times.
- Your blood pressure will be recorded as well.

1. Your blood pressure response to sustained hand- grip will be checked using a hand dynamometer with the dominant hand.

- Your will be asked to grip the equipment at your maximum grip strength and it will be recorded 3 times and average will be taken(Tmax).
- Then you will be asked to grip at 30% of the maximum grip strength(Tmax) and sustain the grip for 5 minutes.
- Blood pressure will be measured three times on the opposite arm before handgrip and 3-4 times per min during the test.
  - 1. Date and time of the appointment_____________________________________________

**Measurement of gut transit time**

- This is measured using lactulose hydrogen breath test using hydrogen breath analyzer.


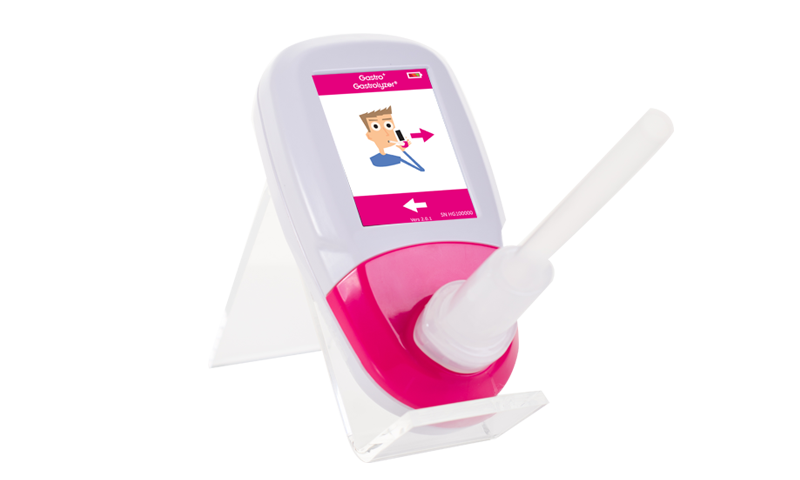


- You will be asked to come overnight fasting for food and milk.

Hydrogen breath analyser

- You will be asked to inhale fully and breath out into the breath analyser.
- A liquid including 10 g of lactulose (a laxative) in 100 mL of water will be given to drink.
- After 1o minutes you will be asked to exhale to the breath analyser. This will be done in 10minute intervals till the cut- off value of hydrogen ≥10 ppm is reached and followed by at least two other subsequent rise.
- This test will take minimum of 40 minutes to maximum of 2 hours.
- You can drink water during the study.
- If you become hungry or your sugar level drops, we will give dextrose solution to drink to correct the sugar level in your body.
- After completion of the test you will be given breakfast.
- Only side effect will be that you may get diarrhoea after the test as lactulose is a laxative. It is not absorbed by the body hence there are no other side effects.

**Meditation program**

Date of commencement _________________________

Dates of follow up sessions_______________________

- This program is based on Buddhist teaching but the actual program does not have any content of the Buddhist preaching nor you will be asked restrict your normal behaviour.
- Aim is for the participants to practice mindfulness (keeping the attention in the present moment) techniques and achieve state of calmness and relaxation of the mind.
- There will be one-hour introductory lecture by a qualified meditation instructor which will be followed by personal interview.
- You will be expected to attend once a week 30-minute sessions where mindfulness practice sessions and meditation will be taught.
- You will be asked to walk for 5 minutes with keeping your attention only to your feet/sensation of the feet. If you get distracted you are expected to again focus on the present moment that is walking.
- Then you will be asked to sit on a chair mindfully (focusing on the posture of your body) for 10 minutes.
- Then you will be asked to focus your attention on to the tip of your nose or to the chest where you can feel the breath while quietly breathing.
- You will be asked to focus the attention to the feeling of your breath calmly and to feel inhalation and exhalation of breath for 15-20 minutes.
- Several other techniques to focus your attention will also be taught. Examples listening to a bell, mindful eating, etc.
- You will also be given home assignments for 12 weeks which will only take 20-30 minutes each day to practice.
- These will be mainly aiming at continuing practising of meditation at home.
- You will be given a diary to record your meditation practices done at home.
- You will be allowed to ask questions/clarify your doubts during the training sessions.
- If you feel you are not able to participate or find it difficult to carry out these techniques even after instructors help, you may leave the study without any problem.
- You will be given contact details of the instructor on the first day if you need to contact and get more details or if you need any clarifications.
