# Supplementary material for: Effects of meditation on physiological and metabolic parameters in patients with type 2 diabetes mellitus “MindDM”: study protocol for a randomized controlled trial
Source: Trials. 2022 Sep 30;23:821. doi: 10.1186/s13063-022-06771-2 (PMC9523920; doi:10.1186/s13063-022-06771-2)
Supplement: Supplementary file 3 — Additional file 3. [file 13063_2022_6771_MOESM3_ESM.docx]

Effects of meditation on physiological and metabolic parameters in patients with type 2 diabetes mellitus.

”MindDM”

**CONSENT FORM**

**Part A - To be filled by the participant**

The participant should complete the whole of this sheet herself.

1. Have you read the information sheet? (Please keep a copy for yourself) YES/NO
2. Have you had an opportunity to discuss this study and ask any questions? YES/NO
3. Have you had satisfactory answers to all your questions? YES/NO
4. Have you received enough information about the study? YES/NO
5. Who explained the study to you? ………………………………………………………………..
6. Do you understand that you are free to withdraw from the study at any time, without having to give a reason and without affecting your future medical care? YES/NO
7. Information held by the investigators relating to your participation in this study may be examined by other research assistants. All personal details will be treated as STRICTLY CONFIDENTIAL. Do you give your permission for these individuals to have access to your records? YES/NO
8. Have you had sufficient time to come to your decision? YES/NO
9. Do you agree to take part in this study? YES/NO

Participant’s signature: …………………………..………… Date:………………………………….

Name (BLOCK CAPITALS):

………………………………………………………………………………………………………………………………………………………

**Part B - To be filled by the investigator**

I have explained the study to the above volunteer and she has indicated her willingness to take part.

Signature of investigator: ……………………................ Date: ……………………………………………………………….

Name (BLOCK CAPITALS):

………………………………………………………………………………………………………………………………………………………
