# Supplementary material for: Effects of meditation on physiological and metabolic parameters in patients with type 2 diabetes mellitus “MindDM”: study protocol for a randomized controlled trial
Source: Trials. 2022 Sep 30;23:821. doi: 10.1186/s13063-022-06771-2 (PMC9523920; doi:10.1186/s13063-022-06771-2)
Supplement: Supplementary file 4 — Additional file 4. [file 13063_2022_6771_MOESM4_ESM.docx]

Effects of meditation on physiological and metabolic parameters in patients with type 2 diabetes mellitus.

”MindDM”

**INFORMATION SHEET**

I, Dr. Chamila Dalpatadu, am a Senior lecturer and a consultant physician attached to the Department of Physiology, Faculty of Medicine, University of Colombo. I would like to invite you to take part in the research study on how meditation affects the metabolism of human body and how meditation affects diabetes control. This study is a randomized controlled trial supervised by Prof. Priyadarashai Galappatthy, Prof Saroj Jayasinghe and Prof. Prasad Katulanda and will be done at the Faculty of Medicine, University of Colombo. This research is funded by AHEAD 81 grant.

1. **Purpose of the study**

The purpose of this research is to identify how meditation affects the metabolic functions of the human body and the effects on diabetes control.

1. **Voluntary participation**

Your participation in this study is voluntary. You are free to not participate at all or to withdraw from the study at any time despite consenting to take part earlier. There will be no loss of medical care or any other available treatment for your illness or condition to which you are otherwise entitled. If you decide not to participate or withdraw from the study you may do so at any time.

1. **Duration, procedures of the study and participant’s responsibilities**

This study will be conducted over a period of one year. If you volunteer to participate in this study, we will ask you to do the following. You will be randomly allocated to either meditation or waitlisted groups. Meditation group will undergo mindfulness meditation program for first 3 months while the waitlisted group will be requested to participated for a mindful breathing meditation program for which participation is not mandatory.

1. You will need to come to the Faculty of Medicine, University of Colombo once a week for 3 months for the meditation program and once a month subsequently. Prior to starting the study and after 3 months there will be requests for additional visits (maximum 2) for investigations.
2. **Physiological tests**

These tests are lactulose hydrogen breath test to assess the time taken for the food to travel through the gut and autonomic function test to assess the response of the heart and the autonomic nervous system. These will be done at the faculty of Medicine Colombo. Details of each investigation is attached (annex 1)

1. **Blood tests**

You will need to come fasting for 10 hours, avoid coffee, tea and exercises in the morning, for the first visit and after 3 months.

1. We will collect 10 millilitres of blood on first visit and after 3 months. Blood is collected for fasting blood sugar, serum fructosamine and HbA1c to check the diabetes control. Furthermore full blood count to check haemoglobin level, serum insulin to check insulin hormone level in the blood as impairment of insulin hormone activity causes diabetes, lipid profile to check cholesterol level will be carried out. Apart from these investigations collected blood will not be used for any other investigation nor any genetic testing.
2. Urine will be collected for 24hour period and 24-hour urine cortisol will be checked, at first visit and after 3 months as excess cortisol hormone leads to poor diabetes control.
3. Blood pressure will be measured seated and standing and an ECG will be done on the first day and after three months.
4. During the study period your routine clinic visits will remain as usual. Doctors at the clinic may request investigations not related to this study depending on your condition.
5. **Mindful breathing meditation program(details in annex 1)**

Meditation program is conducted by an experienced instructor and it is a simple technique that everyone can practice. It is not related to any religious belief or ritual.

There will be a one-hour introductory lecture followed by personal interview with an experienced instructor

You will have to attend once a week 30minutes meditation session.

Home assignments will be given for a period of 12 weeks to be carried out daily for 30 minutes.

You are expected to maintain a diary regarding the meditation assignment done at home and bring the diary to the weekly meditation session.

1. **Potential benefits**

Participation in this study may benefit you by screening for hypertension, lipid (cholesterol) levels and anaemia.

1. **Risks, hazards and discomforts**

We will collect blood samples from you (15 millilitres on two occasions) using a needle and a syringe by an experienced nurse or technical officer which usually causes minimal pain. Blood will be used only for the above mentioned investigaitons.

1. **Reimbursements**

You would be paid a sum of Rs. 500/= per each visit for the travelling expenses.

1. **Confidentiality**

Confidentiality of all records is guaranteed and no information by which you can be identified will be released or published. These data will never be used in such a way that you could be identified in any way in any public presentation or publication without your express permission.

1. **Termination of study participation**

You may stop participating in this study at any time with no penalty. Please notify the investigator as soon as you decide to withdraw your consent. At the time of withdrawal from the study permission would be taken from you prior to using any data that is already collected.

1. **Clarifications**

If you have questions about any of the tests / procedures or information, please feel free to ask the person listed below.

- Dr. Chamila Dalpatadu

Senior Lecturer, Department of Anatomy, Faculty of Medicine, University of Colombo

0713223378

chamila@physiol.cmb.ac.lk

**This project has been approved by the Ethics Review Committee, Faculty of Medicine, University of Colombo. You may contact the committee if you wish to seek clarifications, record any concerns or make complaints about the study by calling 0112695300 extension 240 (between 9am and 4pm) or by sending an email to** [**info.ethics@med.cmb.ac.lk**](mailto:info.ethics@med.cmb.ac.lk)
